# The association of glutathione S-transferase *GSTT1* and *GSTM1* gene polymorphism with pseudoexfoliative glaucoma in a Pakistani population

**Published:** 2010-10-26

**Authors:** Muhammad Imran Khan, Shazia Micheal, Farah Akhtar, Waqar Ahmed, Bushra Ijaz, Asifa Ahmed, Raheel Qamar

**Affiliations:** 1 Department of Biosciences, COMSATS Institute of Information Technology, Islamabad, Pakistan; 2 Al-Shifa Trust Eye Hospital, Rawalpindi, Pakistan; 3 Shifa College of Medicine, Islamabad, Pakistan

## Abstract

**Purpose:**

The aim of the present study was to investigate the association of glutathione S-transferase *GSTT1* and *GSTM1* genotypes with pseudoexfoliative glaucoma (PEXG) in a group of Pakistani patients.

**Methods:**

Multiplex polymerase chain reaction was used to study the *GSTT1* and *GSTM1* polymorphisms in 165 PEXG patients and 162 unaffected controls.

**Results:**

In the current study we describe a significant gender-specific association of *GSTT1* and *GSTM1* null genotypes with PEXG. The three null genotype combinations (i.e., T1M0, T0M1, and T0M0) were found at significantly higher frequencies in the PEXG patients as compared to the controls (χ^2^=21.82, p<0.001). This association was specifically related to the female patients (χ^2^=35.63, p<0.001); no such association was seen in the male patients (χ^2^=2.28, p>0.05).

**Conclusions:**

The results suggest that there is a significant involvement of the *GSTT1* and *GSTM1* polymorphisms in female Pakistani patients having PEXG, which suggests a possible gender-specific impairment of detoxification in this group.

## Introduction

Xenobiotic compounds of exogenous and endogenous origin are a substantial threat to the human cells as they lead to the production of highly reactive oxygen species (ROS). The cells produce numerous antioxidants that counter the effects of these compounds by reducing their accumulation. Glutathione (GSH) is an important antioxidant that protects against cellular damage caused by environmental toxins as well as from ROS-mediated injury. GSH works by neutralizing ROS and xenobiotics with the help of glutathione S-transferase (GST); this enzyme catalyzes the conjugation of these compounds with GSH, which being water soluble can thus be easily eliminated from the body [1-3].

Mammalian GSTs are made up of a supergene family of catalytic and binding proteins located on at least seven different genes, which are divided into three major classes: cytosolic, mitochondrial and microsomal GSTs. Tissue expression studies have shown that most of the cytosolic GSTs are expressed in the kidneys and the liver, where they play an important role in the detoxification of various endogenous and exogenous toxic chemicals in the body [4-6].

Of the cytosolic GSTs, the Mu (*µ*), Theta (σ), and Pi (π) genes have been found at different frequencies in various ethnic groups. In the Mu class of GSTs the M1 null genotype (M0) is common in the Chinese, Japanese, French, and English, with a frequency between 43% and 58% [7-11]. In the Theta class of GSTs the T1 null genotype (T0) has been found at varying frequencies in different ethnic groups: 64.4% in Chinese, 60.2% in Koreans, and 20%–24% in African-Americans [12]. Biochemical studies have associated these allelic variations to intra-individual differences in the ability to metabolize environmental and cellular toxins [13].

It has been shown that individuals carrying the null genotypes of GST may have higher levels of intermediates of oxidative metabolism because the detoxification pathways have been disrupted, and this then directly or indirectly exacerbates the pathological effects of ROS.

This has important implications in a range of diseases; for example, various types of cancers, asthma and others, and has also demonstrated involvement in causing neuronal cell death in neurodegenerative diseases, such as Alzheimer, motor neuron disease and Parkinson [14-17]. GSTs have also been reported to be widely expressed in different ocular tissues. Thus, for individuals carrying the null genotypes, the body’s defense against oxidative damage may be impaired, contributing to manifestation of the ocular diseases in question [18-23].

Polymorphisms of GST have previously been shown to be associated with glaucoma, cataract, exudative age-related macular degeneration as well as various spontaneous optic neuropathies [13,24-27].

Several studies have been conducted in different populations to determine the association of *GSTT1* and *GSTM1* polymorphisms with primary open-angle glaucoma [13,26,28], but to date only three studies have been reported on pseudo-exfoliative glaucoma (PEXG) in populations of Arabs, Turks and Swedes [29-31]. There was no significant association found between PEXG and the null genotypes of *GSTT1* and *GSTM1* in the Turks and Swedes, whereas in the Arab glaucoma patients (n=107), in a study that included POAG, PCAG and PEXG, a significant association of all the deletion genotypes was observed [29]. However, after stratification of patients by glaucoma type the T0M0 genotype was not found to be significantly associated with any type of glaucoma.

The aim of the present study was to determine if there was a significant association of the *GSTT1* and *GSTM1* polymorphisms with PEXG in a Pakistani cohort. The study was approved by the Departmental Ethics Committee of COMSATS Institute of Information Technology, Islamabad and the relevant Hospitals’ Ethics Committee and conformed to the principles of the Declaration of Helsinki. Informed written consent was taken from all patients and unaffected control individuals before sampling. PEXG patients were recruited from the out-patients Department of the Al-Shifa Trust Eye Hospital, Rawalpindi and Christian Eye Hospital, Taxila.

## Methods

### Criteria for patient selection, sample collection and DNA extraction

Complete ophthalmic examinations were performed on the PEXG patients, including measurement of cup-to-disk ratio, tonometeric assessment of intra-ocular pressure and slit lamp biomicroscopy was performed to detect any presence of exfoliative material along the papillary border and on the iris. Following this, the pupils of the patients were dilated and the anterior of the lens surface was examined for any deposits of white material. Angles were measured in all the patients with the help of gonioscopy. All of the healthy control individuals were also examined and they were found to have normal visual fields, no exfoliation material in the eye or any other evidence of glaucoma. Blood samples from all the patients and controls were collected by venipuncture; genomic DNA was extracted by a conventional phenol chloroform method, as described previously [32].

### Genotype analysis of *GSTT1* and *GSTM1* polymorphisms

To determine the *GSTT1* and *GSTM1* genotypes of the subjects, multiplex polymerase chain reaction (PCR) amplification was performed using the following primers: *GSTT1* forward primer 5′-TTC CTT ACT GGT CCT CAC ATC TC-3′; *GSTT1* reverse primer 5′-TCA CCG GAT CAT GGC CAG CA-3′; *GSTM1* forward primer 5′-GAA CTC CCT GAA AAG CTA AAG C-3′; and *GSTM1* reverse primer 5′-GTT GGG CTC AAA TAT ACG GTG G-3′. Beta globin gene sequence amplification was used as an internal control in the PCR reactions, for which the primers were: forward primer 5′-CAA CTT CAT CCA CGT TCA CC-3′; reverse primer 5′-GAA GAG CCA AGG ACA GGT AC-3′. Each 25 μl PCR reaction contained 1× Taq Buffer (10 mM Tris-HCl, pH 9.0, 50 mM KCl, 0.1% Triton X-100, 0.01% gelatine; Fermentas, Burlington, Ontario), 30 pmol of each primer, 1.5 mM MgCl_2_, 0.3 mM dNTP, 1.5 U of Taq DNA polymerase (Fermentas) and 100 ng of genomic DNA.

**Figure 1:**
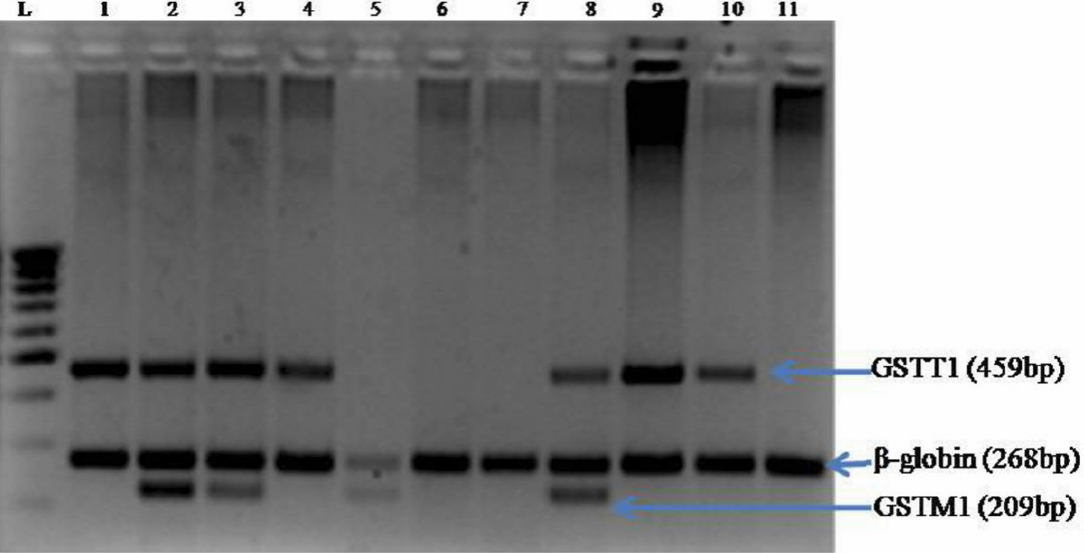
Multiplex PCR amplification product of the *GSTT1*, *GSTM1* and internal control β-globin genes. The amplified products were separated by electrophoresis on 2% agarose gel. Lane L, 100bp DNA ladder; lane 1, 4, 9, and 10, T1/M0 genotype (459 bp and 268 bp fragments); lane 2, 3, and 8, T1/M1 genotype (459 bp, 268 bp, and 209 bp fragments); lane 5, T0/M1 genotype (268 bp and 209 bp fragments); lane 6, 7, and 11, T0/M0 genotype (268 bp fragments).

Amplification was performed with initial denaturation at 95 °C for 5 min, followed by 30 cycles at 95 °C for 1 min, 65 °C for 1 min and 72 °C for 1 min, with a final extension at 72 °C for 7 min. PCR products were electrophoretically separated on 2% agarose gels and the bands were visualized by UV transillumination. For the T1M1 genotype three bands were obtained: a 459 bp band of *GSTT1*, a 209 bp band of *GSTM1* and a 268 bp band of the internal control (β-globin gene; Figure 1). The T1M0 genotype produced two bands of 459 bp and 268 bp; the T0M1 genotype produced two bands of 209 bp and 268 bp. In the case of the T0M0 null genotype only, the β-globin gene internal control band (268 bp) was observed. To confirm the null results, confirmatory PCR tests were performed separately for the *GSTT1* as well as the *GSTM1* genotypes, using β-globin gene amplification as the internal control, under identical conditions as those described above, but using only the *GSTT1* and β-globin or the *GSTM1* and β-globin primers, respectively.

### Statistical analysis

Statistical analysis of the genotype frequencies of both the PEXG patients and controls was performed using the chi-square test (χ^2^). To prevent any false positive inference, Bonferroni correction (p′_b_) was applied to the genotype data. Correction involved multiplying the p value obtained after each single test with the total number of independent tests (k) performed during the study [33,34]. The Sidak correction (p′_s_), an approximation of Bonferroni, was also applied to correct the p value as some Bonferroni corrected values were >1 [35,36]. Note that for the Bonferroni corrected values, the level of significance remained 0.05; i.e., the association of any genotype group/variable with k×p<0.05 was considered as statistically significant [33]. The formulae for the calculation of the Bonferroni and Sidak values are as follows:

$$\text{p}\prime_{\text{b }}=\text{ k }\times \text{ p}$$

$$\text{p}\prime_{\text{s }}=\text{ 1}-{\left(\text{1}-\text{p}\right)}^{\text{k}}$$

where k is the number of genotype groups tested and p is the raw value obtained from the χ^2^ test.

All analyses were performed using SPSS v.16 statistical analysis software (SPSS Inc., Chicago, IL) and StatCalc EpiInfo package v.6 (Atlanta, GA).

## Results

The case-controlled study included 165 patients with glaucoma (53% males, mean age 45.8±10.1 years and 47% females, mean age 46.31±11.6 years), as well as 162 unaffected controls (52% males, mean age 43.8±13.9 years and 48% females, mean age 43.1±10.9 years); there was no statistically significant difference (p>0.05) in the mean age of male/female patients and controls.

There was a significant difference in the overall distribution of the *GSTT1* and *GSTM1* genotypes in the PEXG patients and controls (χ^2^=21.82, p<0.001; Table 1). The difference between individual genotypes remained significant even after the application of Bonferroni and Sidak corrections (p’_b_ & p’_s_<0.05). When the subjects were stratified according to gender the overall genotype distribution of the female patients was found to be significantly associated with the disease (χ^2^=35.63, p<0.001; Table 1); this remained significant even after applying the Bonferroni correction (p’_b_ & p’_s_<0.001). Interestingly, the T0M0 null genotype was exclusively present in the PEXG female patients (χ^2^=20.12, p, p’_b_ & p’_s_<0.001). In the male patients 8.2% of the controls had the T0M0 null genotype as compared to 6% of the PEXG male patients (χ^2^=0.11, p, p’_b_ & p’_s_>0.05, OR=0.81 [95% CI=0.20–3.24]; Table 1).

**Table 1:** Overall and gender segregated data of *GST* genotypes in unaffected controls and PEXG patients.

**Group**	**Genotype**	**Controls**	**Patients**	**p (χ^2^)**	**p (χ^2^)**	**p_b_**	**p_s_**	**OR (95% CI)**
Total	T1M1	95 (59%)	57 (34.5%)	<0.001 (21.82)	Reference			
	T1M0	51 (31%)	69 (41%)		0.001 (10.79)	<0.05	<0.05	2.25 (1.34–3.79)
	T0M1	9 (6%)	23 (14%)		<0.001 (12.71)	<0.001	<0.001	4.26 (1.73–10.74)
	T0M0	7 (4%)	16 (9.5%)		<0.05 (8.45)	<0.05	<0.05	3.81 (1.37–10.96
	Total	162	165					
Females	Genotype	Controls	Patients	p (χ2)	p (χ2)	pb	ps	OR (95% CI)
	T1M1	60 (80.5%)	26 (33.8%)	<0.001 (35.63)	Reference			
	T1M0	16 (20.8%)	31 (40.2%)		<0.001 (15.84)	<0.001	<0.001	4.47 (1.96–10.29)
	T0M1	1 (1.2%)	9 (11.7%)		<0.001 (13.81)	<0.001	<0.001	20.77 (2.45–460.38)
	T0M0	0 (0%)	11 (14.3%)		<0.001 (20.12)	<0.001	<0.001	N/A
	Total	77	77					
Males	Genotype	Controls	Patients	p (χ2)	p (χ2)	pb	ps	OR (95% CI)
	T1M1	35 (41.2%)	31 (35%)	>0.05 (2.28)	Reference			
	T1M0	35 (41.2%)	38 (43%)		>0.05 (0.36)	>0.05	>0.05	1.23 (0.60–2.52)
	T0M1	8(9.4)	14 (16%)		>0.05 (1.83)	>0.05	>0.05	1.98 (0.66–6.01)
	T0M0	7 (8.2%)	5 (6%)		>0.05 (0.11)	>0.05	>0.05	0.81 (0.20–3.24)
	Total	85	88					

pb indicates Bonferroni corrected value and ps indicates Sidak corrected value. A p<0.05 was considered to be statistically significant.

The gender distribution of the null genotypes GSTT0 and GSTM0 were also compared between patients and contols; these genotypes were found at a significantly higher frequency in the female patients than in the female controls (χ^2^=19.90, and χ^2^=18.7, respectively, p<0.05; Table 2); whereas in males the frequency of the null genotypes did not differ significantly between the groups (χ^2^=0.43, χ^2^=0.01, respectively, p>0.05). However, the male control samples as compared to the female controls had a significantly higher frequency of both the null genotypes GSTT0 and GSTM0 (χ^2^=12.13, χ^2^=14.41, respectively, p<0.001; Table 3), whereas in patients in both these groups these genotypes were not statistically different from each other (χ^2^=0.44, χ^2^=0.53, respectively, p>0.05).

**Table 2:** Comparison of *GSTT1* and *GSTM1* null genotypes according to gender distribution in patients and controls. A p<0.05 was considered statistically significant.

**Genotype**	**Group**	**Controls (n=162)**	**Patients (n=165)**	**p (χ^2^)**
T0	Total	16 (10%)	39 (24%)	<0.05 (11.06)
T1		146 (90%)	126 (76%)	
M0	Total	58 (36%)	85 (52%)	<0.05 (8.20)
M1		104 (64%)	80 (48%)	
Genotype		Controls (n=77)	Patients (n=77)	p (χ^2^)
T0	Females	1 (1%)	20 (26%)	<0.05 (19.90)
T1		76 (99%)	57 (74%)	
M0	Females	16(21%)	42(55%)	<0.05 (18.70)
M1		61(79%)	35(45%)	
Genotype		Controls (n=85)	Patients (n=88)	p (χ2)
T0	Males	15(18%)	19(22%)	>0.05(0.43)
T1		70(82%)	69(78%)	
M0	Males	42(49%)	43(49%)	>0.05(0.01)
M1		43(51%)	45(51%)	

**Table 3:** Comparison of *GSTT1* and *GSTM1* null genotypes between male and female controls and patients. A p<0.05 was considered statistically significant.

	**Controls**			**Patients**		
**Genotypes**	**(Males) (n=85)**	**(Females) (n=77)**	**p (χ^2^)**	**(Males) (n=88)**	**(Females) (n=77)**	**p (χ^2^)**
GSTT1 null	15 (18%)	1 (1%)	<0.001 (12.13)	19 (22%)	20 (26%)	>0.05 (0.44)
GSTM1 null	42 (49%)	16 (21%)	<0.001 (14.41)	43 (49%)	42 (55%)	>0.05 (0.53)


## Discussion

Oxidative stress along with cellular senescence is one of the major factors affecting cellular processes. The inability of the cells to cope with oxidative stress is due to a breakdown of the body’s antioxidant defenses due to excessive production of ROS; this in turn leads to damage to the cellular macromolecules, including DNA, proteins and lipids. To counteract the stress-induced damage, the cells upregulate antioxidant enzymes, such as GST [37].

In the eye a system of trabecular meshwork (TM) regulates the outflow of aqueous humor, and maintains normal intraocular pressure (IOP); therefore, any challenge to the structural and functional integrity of TM results in the development of glaucoma. Several lines of evidence suggest that one effect of the generation of oxidative free radicals in the glaucomatous eye is the progressive loss of TM [38]. In an experimental rat model of glaucoma the retinal ganglion cells (RGC) and glial cells showed extensive protein and lipid oxidation, which appears to lead to apoptosis of these and other neuronal cells [37,39]. Thus, to maintain the homeostatic balance between the production of ROS and their clearance, the eye, under stress, is required to produce higher levels of antioxidants. Indeed, the glaucomatous eye upregulates several stress-related genes, including GST, which are involved in the detoxification of ROS and other related compounds [39,40].

Although, under stress, GST and other antioxidants are demonstrably upregulated, the glaucomatous eye fails to prevent oxidative damage, a fact that points to an underlying molecular or genetic defect. One such genetic risk factor is the presence of the GST null genotype in patients having oxidative, stress-related diseases.

A potent antioxidant 8-hydroxy-2′-deoxyguanosine (8-OH-dG) has been shown to be present at 3.6 fold higher levels in the TM of POAG patients, as compared to controls [41]. In these patients there was a positive correlation between oxidative DNA damage and intraocular pressure as well as visual field defects. The *GSTT1* null genotype was found to be more common in POAG cases, who also showed 2.2 fold higher levels of 8-OH-dG as compared to the other genotypes [41,42], resulting in oxidative DNA damage in the TM of these patients. A higher level of oxidative damage to the trabecular meshwork has also been seen in POAG patients with the *GSTM1* null genotype, indicating the possible involvement of these genotypes in the manifestation of disease [42].

In primary cultures of human optic nerve head astrocytes from glaucomatous eyes it has been observed that the basal levels of GSH antioxidants were well below those of primary cultures from normal astrocytes; these data combined with all the other evidence indicates that oxidative stress plays a significant role in the manifestation and progression of glaucoma [40].

Recently, using cDNA arrays, the involvement of several stress-related candidate genes was studied in PEXG. The authors found that a large set of cytoprotective gene products, including antioxidant defense enzymes (e.g., GST) and stress-inducible transcription factors, were consistently down-regulated in PEXG at both the mRNA and protein levels; this finding supports the conjecture that GSTs play an important role in protecting the eye against the development of PEXG [41].

The present study was based on the hypothesis that inadequate expression of GST in PEXG correlates with the null genotypes. This hypothesis has been tested in Swedes [31] and Turks [28], but in those populations no significant contribution of the polymorphic variants of GSTs with PEXG was found. To the best of our knowledge, the investigators in all of those studies did not stratify their data according to gender. This may be significant considering our knowledge that in mice there is a higher expression of some types of GSTs in females as compared to males [6]. This indicates that any defects in the relevant genetic pathways in females could exacerbate the risk of disease to a significantly higher level than for males.

The importance of the present study is that an association between PEXG and all the GST null genotype combinations was observed and this remained significant even after Bonferroni correction. In addition, after stratifying the data according to gender, a clear association of the null genotypes with only the female PEXG patients was found; this occurred because a large number of female PEXG patients were found to carry the null genotypes, in different combinations, as compared to the female controls (Table 1).

The *GSTT1* and *GSTM1* null genotypes in the total data set of the patient cohort was also compared, as well as in the data stratified according to gender. Although an overall statistically significant difference in the null genotype distribution between patients and controls was observed, this was due to the significantly higher distribution of the null genotypes in the female patients, as compared to female controls (Table 2). In addition it must be pointed out that the significantly higher frequency of the T1 and M1 in the female unaffected controls as compared to the males (Table 3) could be a result of a gender-specific protective effect of these genotypes in the females only.

The association of the *GSTM1* null genotype with female PEXG patients in the current study is in accordance with the data of a group of Greek patients with multiple sclerosis [42]; using this data we calculated and compared the gender-wise distribution of the null genotypes of *GSTT1* and *GSTM1* between the Greek controls and patients. We found that the female patients had a significantly higher frequency of the *GSTM1* null genotype (65.5% versus 41.7% in controls; χ^2^=4.91, p=0.02) while in males there was no difference in the distribution of any of the null genotypes.

Another interesting aspect of the present study is that, in addition to the *GSTM1* null genotype, the frequency of the *GSTT1* null genotype in the female PEXG patients occurred also at a significantly higher level than in the control females (Table 2), while in the Greeks there was no significant difference in this group (χ^2^=0.05, p=0.82). Also, in contrast to our data, in the Greeks the T0M0 combined null genotype was not found to be associated with the females, which points to the possible association of the GST T0M0 genotype with PEXG only in the Pakistani female patients; the resultant vulnerability to cellular and oxidative stress conditions may therefore be a contributing factor in the disease pathology that possibly has ethnic as well as gender associations.

In conclusion, we would like to emphasize the importance of conducting further studies in different populations to further ascertain the association of GST with PEXG; this will allow the development of a consensus regarding the involvement of GST in glaucoma. There is also a need to better understand the mechanisms associated with the null genotypes in female patients.
